# Supplementary figures and images for: Assessing electrocardiogram changes after ischemic stroke with artificial intelligence
Source: PLoS One. 2022 Dec 27;17(12):e0279706. doi: 10.1371/journal.pone.0279706 (PMC9794063; doi:10.1371/journal.pone.0279706)

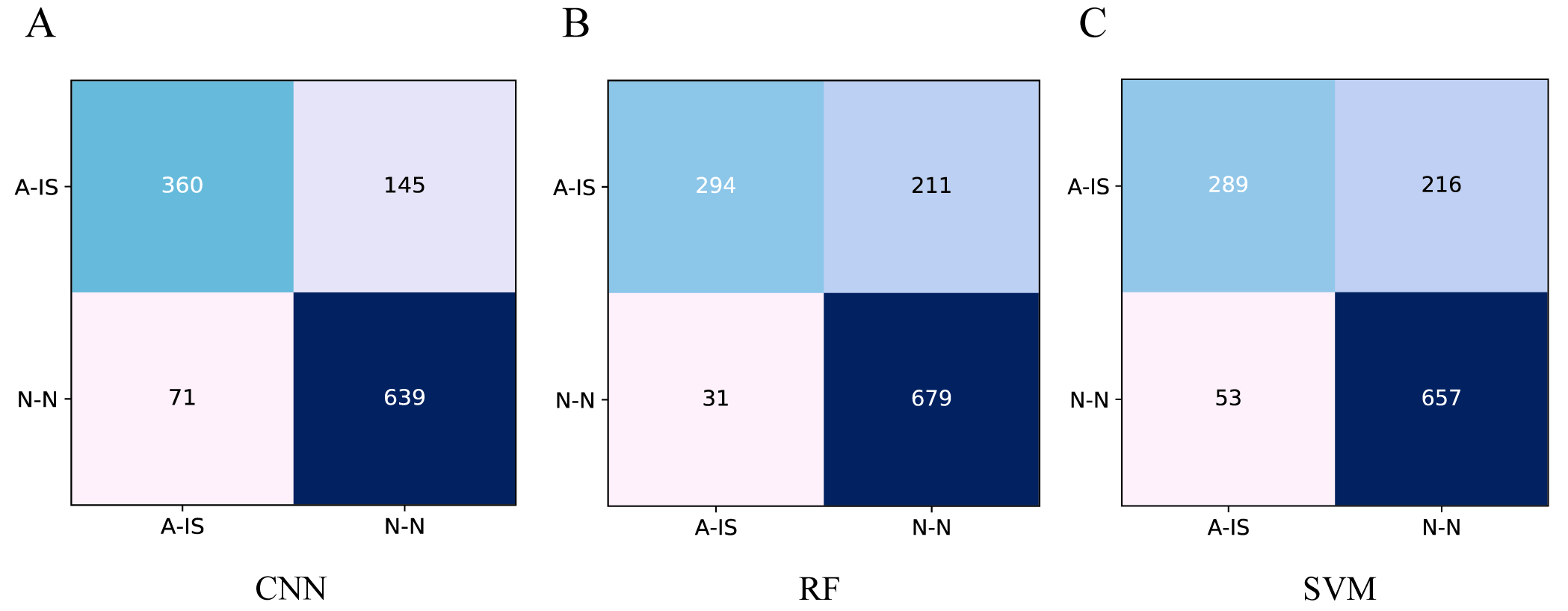

Supplement: S1 Fig — The label on the left is the true label, and the label below is the predicted label. (A) The confusion matrix of the CNN model shows the absolute numbers of classifications made for patients belonging to the A-IS group and N-N group. (B) The confusion matrix of the RF model shows the absolute numbers of classifications made for patients in the A-IS and N-N groups. (C) The confusion matrix of the SVM model shows the absolute numbers of classifications made for patients in the A-IS and N-N groups. (TIF) [file pone.0279706.s001.tif]

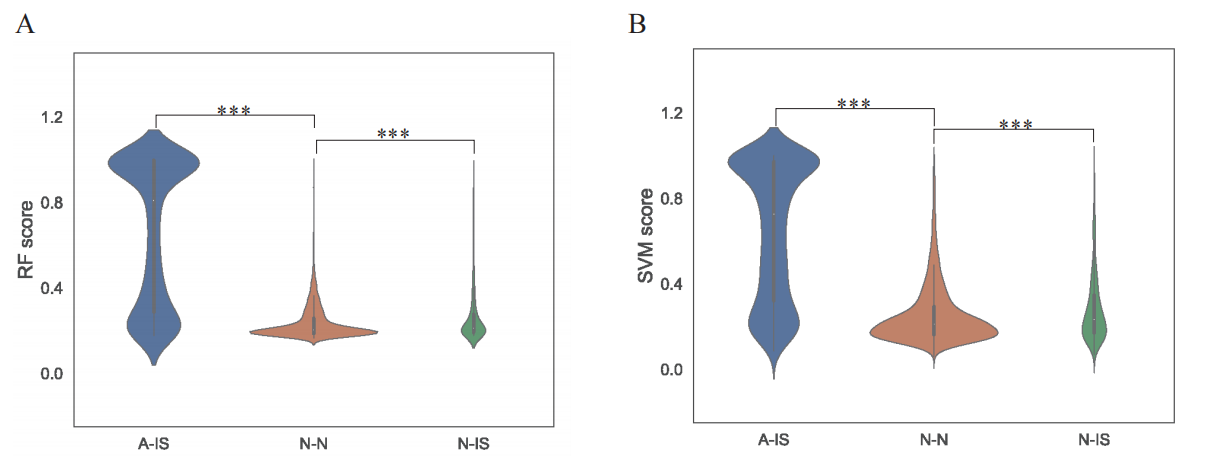

Supplement: S2 Fig — (A) The score distribution of the N-N, N-IS and A-IS datasets was evaluated by the RF model. The scores between the N-N and N-IS datasets are significantly different (p < 0.001, Wilcoxon rank-sum test), as are the scores between the A-IS and N-IS datasets. (B) The score distribution of the N-N, N-IS and A-IS datasets was evaluated by the SVM model. The scores between the N-N and N-IS datasets are significantly different (p < 0.001, Wilcoxon rank-sum test), as are the scores between the A-IS and N-IS datasets. (p < 0.001, Wilcoxon rank-sum test). Note: p < 0.05, *; <0.01, **; <0.001, ***. (TIF) [file pone.0279706.s002.tif]

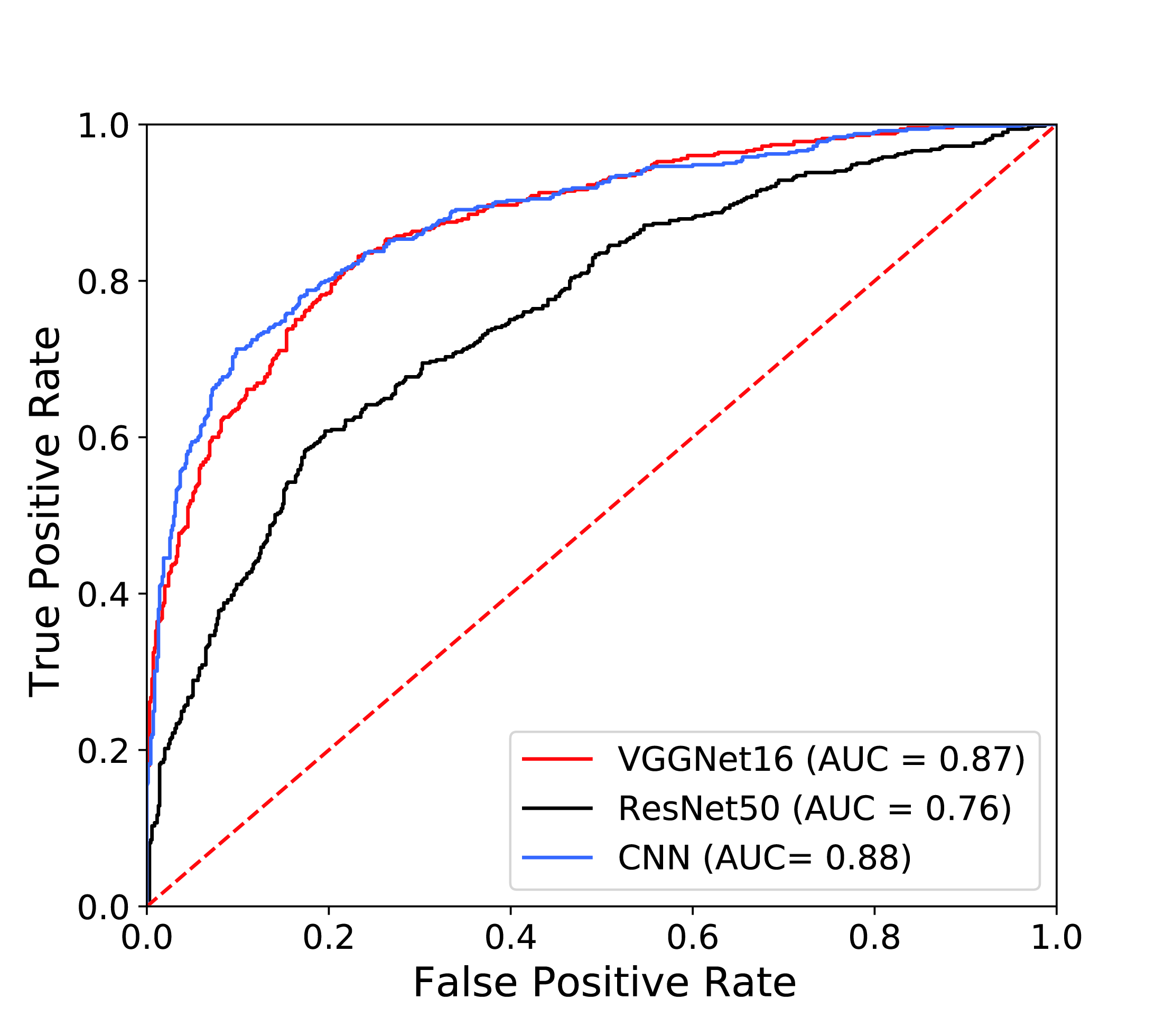

Supplement: S3 Fig — (TIF) [file pone.0279706.s003.tif]
