# Supplementary material for: Assessing electrocardiogram changes after ischemic stroke with artificial intelligence
Source: PLoS One. 2022 Dec 27;17(12):e0279706. doi: 10.1371/journal.pone.0279706 (PMC9794063; doi:10.1371/journal.pone.0279706)
Supplement: S1 Table — (DOCX) [file pone.0279706.s004.docx]

**S1 Table. Network structure and hyperparameters of the CNN model.**

| CNN Architecture | **Size** | **Number of kernel** | **Stride** | **Note** |
| --- | --- | --- | --- | --- |
| **Convolutional layer1** | 11*11 | 128 | 3 | Padding=SAME |
| **Pooling layer1** | 5*5 | / | 3 | Maxpooling |
| **Convolutional layer2** | 5*5 | 256 | 3 | Padding=VALID |
| **Polling layer2** | 3*3 | / | 2 | Maxpooling |
