# Supplementary material for: Assessing electrocardiogram changes after ischemic stroke with artificial intelligence
Source: PLoS One. 2022 Dec 27;17(12):e0279706. doi: 10.1371/journal.pone.0279706 (PMC9794063; doi:10.1371/journal.pone.0279706)
Supplement: S2 Table — (DOCX) [file pone.0279706.s005.docx]

**S2 Table.** ***P* values for the ECG parameters.**

|  | **Heart rate** | **P wave** | **P-R interval** | **QT interval** | **QTc interval** | **QRS wave** | **RV5+SV1** |
| --- | --- | --- | --- | --- | --- | --- | --- |
| ***P_a_*** | <0.001 | <0.001 | 0.34 | <0.001 | <0.001 | <0.001 | 0.046 |
| ***P_b_*** | 0.018 | 0.43 | <0.001 | 0.0013 | 0.14 | 0.11 | 0.20 |

*P_a_*: A-IS dataset *vs.* N-N dataset, *P_b_*: N-IS dataset *vs.* N-N dataset
