# Supplementary material for: Assessing electrocardiogram changes after ischemic stroke with artificial intelligence
Source: PLoS One. 2022 Dec 27;17(12):e0279706. doi: 10.1371/journal.pone.0279706 (PMC9794063; doi:10.1371/journal.pone.0279706)
Supplement: S3 Table — (DOCX) [file pone.0279706.s006.docx]

**S3 Table. Summary of AI model performance.**

|  | **Accuracy** | **Precision** | **Recall** | **F1 score** | **MCC** | **AUC** | **Optimal cutoff value** |
| --- | --- | --- | --- | --- | --- | --- | --- |
| **CNN model** | 0.82 | 0.84 | 0.71 | 0.77 | 0.63 | 0.88 | 0.7669 |
| **RF model** | 0.80 | 0.90 | 0.58 | 0.71 | 0.60 | 0.83 | 0.4212 |
| **SVM model** | 0.78 | 0.85 | 0.57 | 0.68 | 0.55 | 0.82 | 0.4608 |

MCC: Matthews correlation coefficient; AUC: Area Under Curve
