# Supplementary material for: Assessing electrocardiogram changes after ischemic stroke with artificial intelligence
Source: PLoS One. 2022 Dec 27;17(12):e0279706. doi: 10.1371/journal.pone.0279706 (PMC9794063; doi:10.1371/journal.pone.0279706)
Supplement: Attachment — Submitted filename: Response to Reviewers .docx [file pone.0279706.s007.docx]

Dear Editors:

Thank you for your letter comments concerning our manuscript entitled “Assessing electrocardiogram changes after ischemic stroke with artificial intelligence” (PONE-D-22-27898). Those comments are all valuable and very helpful for revising and improving our paper, as well as the important guiding significance to our research. We have studied the comments carefully and made corrections, which we hope meet with approval. Revised portions are marked on the paper. The main corrections in the paper and the responses to the editors are as follows:

**Responds to the Journal’s requirements:**

**(1) Requirements: Please ensure that your manuscript meets PLOS ONE's style requirements, including those for file naming.**

**Response:** Thanks for mentioning the problems. We have checked and modified the article's format according to the magazine's requirements.

**(2) Requirements: Please upload your study’s minimal underlying data set as either Supporting Information files or to a stable, public repository and include the relevant URLs, DOIs, or accession numbers within your revised cover letter.**

**Response:** Thanks for pointing out this problem. We have explained the original data structure in detail in the revised cover letter. All original data we used is available now in the figshare database (<https://doi.org/10.6084/m9.figshare.21523527.v1>; DOI:10.6084/m9.figshare.21523527;) for reproduction.

**(3) Requirements: Your ethics statement should only appear in the Methods section of your manuscript. If your ethics statement is written in any section besides the Methods, please delete it from any other section.**

**Response:** Thanks for your reminder. We have moved the ethics statement from the end of the article to the materials and method section.

**Responses to the reviewers’ comments:**

**Reviewer #1: The contribution is limited given the fact that the proposed work was not compared against state of the art deep-learning methods. In my opinion, a fair literature review should be provided in a separate section; then, a comparison can be made. upon completing this action, the contribution can be fairly jusdged.**

**Response:** Thank you for your advice. We have added some literature review on the current state of CNN applications on ECG analysis in a separate paragraph in the discussion. Also, we input the same data into the Resnet50 network and VGG-16 network (the widely used developed CNN networks) to perform transfer learning, and compared the performances of these two networks with the network used in this study. The results show that there is no significant difference in the performance of the three networks. Also, the convolutional neural network we developed has a simpler structure, which can improve the running speed of the model, to improve the computing efficiency. At the same time, the images we used are ECGs, of which the waveform information needs to be analyzed, so it is unnecessary to analyze the color and other patterns of the picture which requires more deep network to extract enough information to achieve effective training. For example, images like histological slides are input into the network as RGB images which contains a lot of color information, while the electrocardiogram images are input as waveform grayscale images, carrying less information. We developed a CNN model that is more suitable to analysis ECG. We have added this in the discussion section.

**Reviewer #2:**

**(1). No methods have mentioned for extraction of ECG parameter. Kindly, mention the methods which have been used to extract the ECG parameters.**

**Response:** Thank you for your helpful comments. The ECG parameters were generated by the FX-7042 Cardimax ECG machine and recorded in the electronic medical record. Seven ECG parameters, including heart rate, P wave, P-R interval, QRS wave, QT interval, QTc interval, and RV5+SV1 value, were extracted from the electronic medical record and checked manually. We described it in the Material and method section.

**(2). Performance comparison table is missing, kindly add it.**

**Response:** Thank you for your helpful comments. The performance comparison table is in the supporting information (S3 Table. Summary of AI model performance)

**(3). Why only SVM is used in machine learning technique should be clearly mentioned.**

**Response:** Thank you for your helpful comments. In this paper, we have used two machine learning models, the support vector machine model, the random forest model, and one CNN model. CNN performs the best among the three. Unlike CNN which inputs images directly, the machine learning models we built used extracted ECG parameters to perform model training and classification. The weight of each parameter in model development can also be shown as importance, achieving model interpretation to some extent that which parameters are the most important indicators. We have improved this description in the method section.

We tried our best to improve the manuscript and made some changes in the manuscript. These changes will not influence the content and framework of the paper. And here we did not list the changes but marked them in red in the revised paper.

We appreciate for Editors/Reviewers’ warm work sincerely and hope that the correction will meet with approval. Once again, thank you very much for your comments and suggestions.

**Sincerely yours,**

**Libin Deng**

**Professor**

**School of Public Health, Nanchang University; Jiangxi Provincial Key Laboratory of Preventive Medicine, Nanchang University; The Institute of Periodontal Disease, Nanchang University**

**No.461, BaYi Road, Nanchang, Jiangxi Province, P.R.C**

**Tel: +86-15170401580**

**E-mail: lbdeng@ncu.edu.cn**
