# Supplementary material for: Assessing electrocardiogram changes after ischemic stroke with artificial intelligence
Source: PLoS One. 2022 Dec 27;17(12):e0279706. doi: 10.1371/journal.pone.0279706 (PMC9794063; doi:10.1371/journal.pone.0279706)
Supplement: Attachment — Submitted filename: Response to Reviewers .docx [file pone.0279706.s008.docx]

Dear Editors:

Thank you for your letter comments concerning our manuscript entitled “Assessing electrocardiogram changes after ischemic stroke with artificial intelligence” (PONE-D-22-27898). Those comments are all valuable and very helpful for revising and improving our paper, as well as the important guiding significance to our research. We have studied the comments carefully and made corrections, which we hope meet with approval. Revised portions are marked on the paper. The main corrections in the paper and the responses to the editors are as follows:

**Responds to the Journal’s requirements:**

**(1) Requirements: Please review your reference list to ensure that it is complete and correct. If you have cited papers that have been retracted, please include the rationale for doing so in the manuscript text, or remove these references and replace them with relevant current references. Any changes to the reference list should be mentioned in the rebuttal letter that accompanies your revised manuscript. If you need to cite a retracted article, indicate the article’s retracted status in the References list and also include a citation and full reference for the retraction notice.**

**Response:** Thank you for the requirement. We have checked and edited the reference, making sure it meets the criteria of the journal. The details are shown shown in the revised manuscript.

**Responses to the reviewers’ comments:**

**Reviewer #1: All comments have been addressed for the manuscript (Assessing electrocardiogram changes after ischemic stroke with artificial intelligence).**

**Response:** Thank you for your guidance, we are very glad to revise the article under your guidance.

**Reviewer #2:**

**(1). Abstract should be rewritten, as the work is not properly reflected in the abstract.**

**Response:** Thank you for your advice, we have rewritten the abstract.

**(2). In 153 number line authors have written, they have proposed a CNN model. But in presentation of content, it does not look like. It should be changed to they have used CNN model.**

**Response:** Thank you for your comments, we have checked and revised this in the manuscript.

**(3). They have used certain parameters for CNN model, the reason for use of particular parameter has not mentioned. Kindly mention it.**

**Response:** Thanks for pointing out this problem. We adjusted the hyperparameters and structure of the convolutional neural network according to the working speed and performance of the model, based on which we chose the optimal hyperparameters and structure for the model. The details are shown in Method section.

**(4). Authors have written about AuC and ROC, diagram has not given.**

**Response:** Thank you for pointing out this. The figures 1-5 are in the main body of the manuscript. S1-S3 figures are shown in supplemental material. The AUC and ROC diagrams are Figure 3A in the main body and S3 figure in the supplemental material. Here are the titles of the figures.

① Fig 1. Pipeline of AI model development and CNN structure.

② Fig 2. Analysis of participant ECG parameters.

③ Fig 3. Performance of the ML and CNN models.

④ Fig 4. CNN model performance in the validation cohort and in poststroke disability assessment.

⑤ Fig 5. Grad-CAM feature visualization.

⑥ S1 Fig. Confusion matrixes of the three AI models.

⑦ S2 Fig. Score distribution of the N-N, N-IS, and A-IS datasets evaluated by the ML models.

⑧ S3 Fig. ROC curves for Our CNN, VGGNet16, and ResNet50 models.

We tried our best to improve the manuscript and made some changes in the manuscript. These changes will not influence the content and framework of the paper. And here we did not list the changes but marked them in red in the revised paper.

We appreciate for Editors/Reviewers’ warm work sincerely and hope that the correction will meet with approval. Once again, thank you very much for your comments and suggestions.

**Sincerely yours,**

**Libin Deng**

**Professor**

**School of Public Health, Nanchang University; Jiangxi Provincial Key Laboratory of Preventive Medicine, Nanchang University; The Institute of Periodontal Disease, Nanchang University**

**No.461, BaYi Road, Nanchang, Jiangxi Province, P.R.C**

**Tel: +86-15170401580**

**E-mail: lbdeng@ncu.edu.cn**
